# A multiple sclerosis lifestyle behavior online course: Qualitative analysis of participants' motivations, expectations and experiences

**DOI:** 10.3389/fpubh.2022.1022185

**Published:** 2022-12-07

**Authors:** Sandra L. Neate, William Bevens, Pia L. Jelinek, Kathleen M. Gray, T. J. Weiland, Nupur Nag, Steve Simpson-Yap, George A. Jelinek, M. Yu, Jeanette C. Reece

**Affiliations:** ^1^Neuroepidemiology Unit, Centre for Epidemiology and Biostatistics, Melbourne School of Population and Global Health, The University of Melbourne, Melbourne, VIC, Australia; ^2^Centre for Digital Transformation of Health, The University of Melbourne, Melbourne, VIC, Australia; ^3^MS Flagship, Menzies Institute for Medical Research, University of Tasmania, Hobart, TAS, Australia

**Keywords:** digital health, education, lifestyle, multiple sclerosis, qualitative

## Abstract

**Background:**

Modification of lifestyle-related risk factors for multiple sclerosis (MS) has been associated with improved health outcomes when compared with standard medical management alone. Based on an existing lifestyle modification program offered as a residential workshop, the MS Online Course (MSOC) was developed to translate the workshop into an online intervention. We performed a pilot randomized controlled trial (RCT), to assess the feasibility concepts of accessibility, learnability and desirability through quantitative and qualitative analyzes. In the present study, we performed additional qualitative analyzes to explore participants' motivations, expectations, and experiences of the MSOC. This study aims to complement prior feasibility analyzes and inform recruitment strategies and course content redevelopment so that its effectiveness may be assessed by examining behavior change and health outcomes in a future larger RCT.

**Methods:**

Participants were recruited *via* online advertisements and randomized to either: the standard care course, containing material sourced from public facing MS websites; or the intervention course, based on an evidence-based lifestyle modification program for people with MS. Course completers were invited to participate in semi-structured interviews. Within a qualitative paradigm, reflexive thematic analysis of interviews was undertaken.

**Results:**

Of 31 eligible participants, 17 completed the MSOC and 14 agreed to be interviewed. Four themes were identified in this analysis: (1) “Wanting to help others” (helping through volunteering, contributing to knowledge base, spreading the word; (2) “Seeking knowledge” (confirmation of existing knowledge; obtaining new knowledge, relevant, credible information); (3) “Doing what I can to help myself” (understanding lifestyle modification, changing my lifestyle, remaining well); and (4) “Changing attitudes” (finding positivity, feeling more confident and in control).

**Conclusions:**

Participants were motivated to help others through research, help themselves by improving knowledge and to find ways to better manage their MS. Expectations included obtaining credible, reliable information, to substantiate existing knowledge, and to further understand lifestyle modification. Participants' experiences included confirmation of and obtaining new knowledge, and early implementation of modified lifestyle behaviors. These insights surrounding participants' motivations, expectations and experiences will assist in recruitment strategies, course redevelopment and outcome measures for the future RCT to examine the effectiveness of the MSOC.

## Introduction

The treatment landscape of multiple sclerosis (MS) has evolved over recent years. People with MS now describe wellness as a high priority and seek information regarding wellness more frequently than medication-related information (1). Self-management, where individuals take responsibility for their own healthcare related decisions (2), has emerged as part of a new paradigm of MS management. Learning to self-manage health conditions is often enhanced by educational interventions which assist in behavior change to improve health and quality of life outcomes (2).

Modification of lifestyle-related risk factors represents an opportunity for people with MS to play a pivotal role in managing their own health. One lifestyle modification program, the Overcoming MS program (3), which recommends a plant-based whole food diet low in saturated fat, physical activity, vitamin D and omega 3 fatty acid supplementation, smoking cessation and stress reduction, has been associated with reduced relapse rate (4, 5), decreased fatigue (6–8) and depression (9, 10), stabilization of or decreased disability progression, improved quality of life (11–13) and symptom reduction (14, 15). Residential educational workshops delivering the Overcoming MS program (residential workshops) (3) were conducted for many years in Australasia, United Kingdom and Europe. Studies following Australian residential workshop participants demonstrated attendance was associated with improved health-related quality of life (16, 17), reductions in relapse rate and disability (18), and that lifestyle modifications can be sustained (18, 19). Engagement with this program has provided independent benefits to mental health and quality of life (20).

Face-to-face delivery of educational interventions has obvious limitations. Online interventions offer the potential to overcome physical, economic and geographical barriers (21). Online interventions have been developed for several individual aspects of MS management including physical activity (22, 23) fatigue management (24), attention (25), and depression (26) but no studies, to our knowledge, have assessed an intervention which recommends simultaneous modification of multiple risk factors.

Studies of online MS-related educational resources have generally focused on knowledge transfer (27–29). However, interventions that solely provide knowledge may not necessarily result in behavior change and improved health outcomes (30–32). Of the few studies that have examined behavior change, the development of self-efficacy, or the individual's perception that they can influence their outcomes (33), is an important component of effecting change. Education programs where healthcare professionals provide support as well as knowledge are associated with enhanced self-efficacy, decision-making and empowerment in chronic diseases (34). Similarly, engagement with healthcare professionals at residential workshops was associated with an enhanced sense of agency and control (19, 35). Subsequently, an online MS education tool that facilitates both knowledge transfer and important contributors to behavior change, such as self-efficacy, is the ultimate goal.

Our research team developed an online version of the Overcoming MS program previously offered as a residential workshop (3), the Multiple Sclerosis Online Course (MSOC). In order to test the effectiveness of the online course in a randomized controlled trial setting, we developed two courses: a standard care course (SCC) and intervention course (IC). The ultimate aim is to conduct a randomized controlled trial (RCT) that will examine differences in behavior change and health outcomes between the SCC and IC. Prior to conducting this effectiveness RCT we conducted this pilot RCT to quantitatively (36) and qualitatively (37) assess the feasibility of the MSOC. Feasibility domains in the two preceding analyzes (36, 37) were accessibility, learnability and desirability. Participants were randomized to test all elements of the MSOC, although outcomes for feasibility were not expected to significantly differ between arms. The present study reports a second qualitative analysis which aimed to explore participants' motivations for undertaking the MSOC, their expectations of this digital intervention and the outcomes they experienced, to assist in optimizing future recruitment strategies and course content in both arms.

## Methods

The methodology of the RCT and the course development and content have been more fully described previously (36) but are reported here in brief.

### Ethical approval

This study was approved by The University of Melbourne Human Research Ethics Committee (ID: 1851781.2) and was prospectively registered with the Australian New Zealand Clinical Trials Registry (ID: ACTRN12621000245897).

### The multiple sclerosis online course

The MSOC was developed to provide a free, accessible online educational course for people with MS based on the existing evidence-based lifestyle modification program (3). To enable testing of the course, initially in a feasibility-focussed RCT and in a future effectiveness RCT, two arms of the MSOC were developed, the intervention course (IC) and the standard care course (SCC). The content of the two arms was developed by the research team. Both arms were delivered in an asynchronous manner *via* the same website, were identical in format and delivered the same seven modules. Participant profiles and a moderated forum formed part of each arm. The study arms differed only in content.

#### Intervention course

Content was adapted from the Overcoming MS program (3) previously presented as a 5 day residential workshop. Content included an introduction to MS pathophysiology followed by the evidence underpinning the lifestyle recommendations: dietary modification to a plant-based whole food diet plus seafood with omega 3 polyunsaturated fatty acid supplementation; physical activity 20–30 min approximately five times per week; vitamin D supplementation; stress reduction; and prevention in family members.

#### Standard care course

Content was sourced entirely from MS society websites in the public domain, including Multiple Sclerosis Australia, Multiple Sclerosis Research Australia, National MS Society, Multiple Sclerosis Society UK, Multiple Sclerosis Society of Canada and aimed to reflect standard information provided by heath care practitioners and MS societies.

### The feasibility RCT

#### Participant recruitment

Participants were recruited *via* online advertisements through the Canadian, New Zealand, and US MS society websites, and an Australian MS Facebook group. Eighty-four eligible participants were sent baseline surveys, completion of which was required to commence the course and 31 eligible participants were randomized (15 IC, 16 SCC).

**Figure 1:**
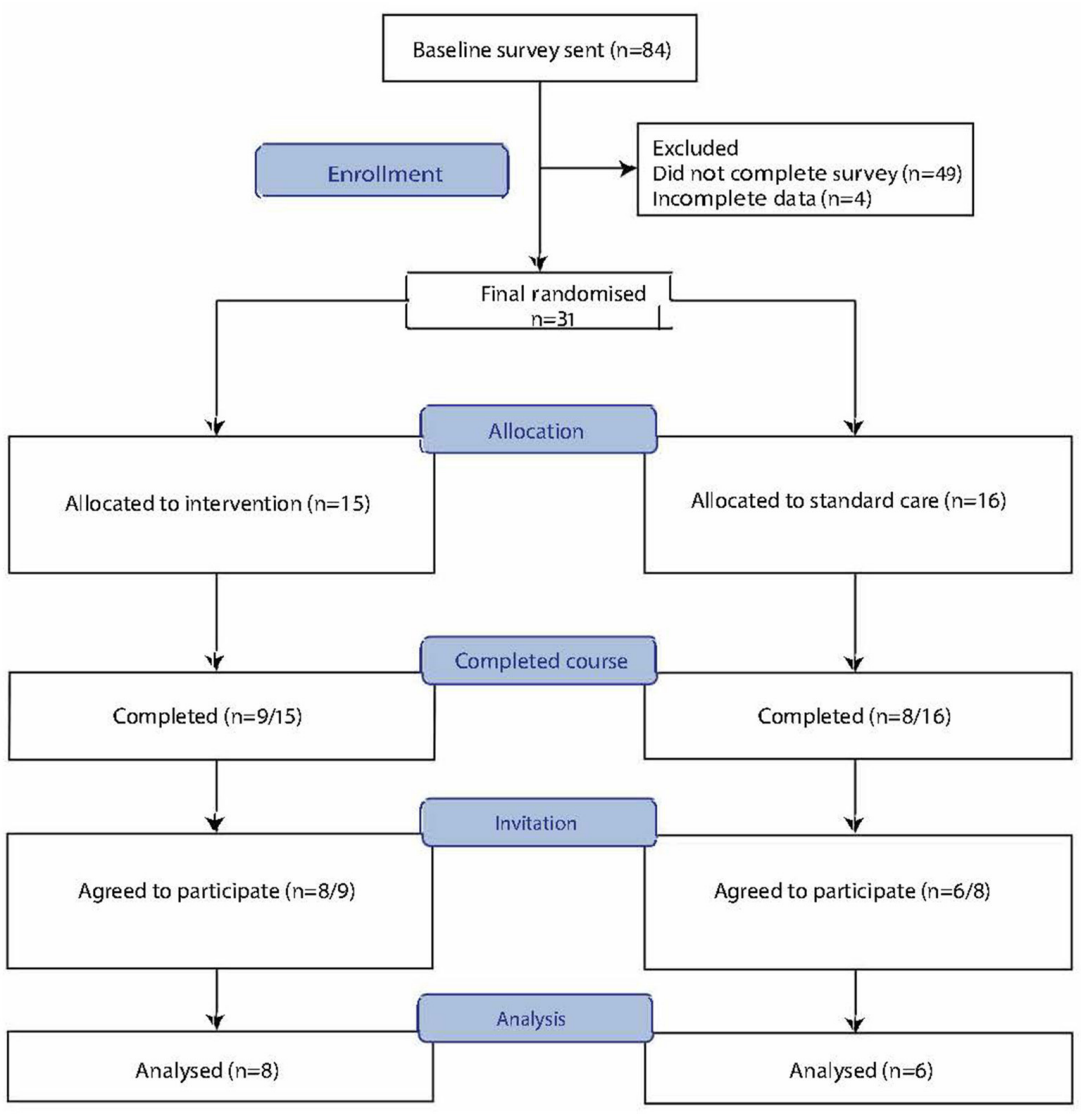
Consort flow diagram of participant recruitment for the multiple sclerosis online course feasibility randomized controlled trial.

17 of these 31 participants (55%) completed the course (nine IC and eight SCC) of whom 14/17 (82% of course completers) consented to participate in semi-structured interviews (eight IC and six SCC) (Figure 1). As feasibility targets (course completion of >40% and >25% in intervention arm and standard-care arms, respectively) were achieved, we did not interview those participants did not complete the courses for these feasibility related analyzes. As the aim of this analysis was to understand participants' motivations, expectations and experiences of the MSOC, participants from both arms were interviewed as it was likely all participants would have feedback regarding these research questions.

Following completion of the MSOC, participation in this qualitative study was requested *via* email.

#### Data collection

The semi-structured interview schedule was developed by researchers to explore motivations, expectations and outcomes experienced (Supplementary Data Sheet 1).

Interviews were conducted by telephone or video-conference (Zoom.us) by three researchers (SN, JR, and PJ) from mid to late June 2021, 2–4 weeks post course completion. Interview duration ranged from 21 to 52 min, average 33.5 min. Interviews were digitally recorded and transcribed de-identified by voice recognition software (temi.com). Interview transcripts were edited and imported into Nvivo software for data management.

#### Data analysis

Within a qualitative paradigm, semi-structured interviews were analyzed using reflexive thematic analysis (38). An inductive approach was used to allow themes to be derived from participants' reflections.

Initial codes were determined and all data extracts from the dataset were collated under the relevant codes (SN) enabling the research team (SN, JR, PJ, and WB) to examine the codes. The data corpus was divided into two datasets. The first dataset included codes related to the explicit feasibility concepts of accessibility, learnability and desirability. The second dataset included codes relating to participants' motivation, expectations and experiences, which were analyzed and reported here.

The second dataset was then re-examined and recoded. IC and SCC interviews were initially coded separately, but researchers noted that the codes were similar between the IC and SCC participants. Researchers identified broader patterns of meaning and generated initial themes. Once again, identified themes were relevant across participants from both courses. The researchers had expected that both codes and themes may be similar across all participants, as we were examining motivations and expectations, attitudes that would have existed prior to course participation. The themes were frequently reviewed and discussed to ensure each theme contained a central organizing concept, reflected a deeper meaning of the data, and were representative across the dataset. Theme names were chosen to represent the reflections of the participants and the coded extracts. Theme names were refined as part of an iterative process. Subthemes were identified, collapsed, and given appropriately descriptive names. Although the themes were relevant across all participants, differences between the SCC and IC cohorts under each theme are described during the analysis and in the discussion, where relevant.

Trustworthiness of the analysis was enhanced by the depth of engagement of researchers with participants during interviews and researchers' immersion in the data. Records of thematic analysis discussions and serial versions of coding and theme development documented the evolution of the analysis. Researchers critically reflected on the analysis and their role in interpretation of the data. Researchers, some of whom had participated in the design and development of the course, acknowledged their subjectivity and the preconceptions they brought to the analysis (39). Verbatim quotes to illustrate themes enhanced the transparency and reliability of data interpretation. The COnsolidated criteria for Reporting Qualitative studies (COREQ): 32-item checklist was utilized (40).

Differences in sample characteristics between the intervention and standard-care courses were assessed by *T*-test and Chi-squared test for continuous and binary/categorical variables, respectively.

## Results

### Participants

The mean age of the 14 participants in this qualitative study was 50 years. The majority in both the IC and SCC were female (88% and 83%, respectively) and diagnosed with relapsing remitting MS (75% and 67%,). Fifty percent IC participants were from the USA, 38% New Zealand, and 13% Australia. In the SCC, participants were from Australia (50%) or NZ (50%). Mean time since diagnosis was 8 and 11 years respectively (Table 1). There were no significant differences in characteristics examined between the IC and SCC participants or between the qualitative participant cohort (*N* = 14) and the initially randomized (*N* = 31) cohort.

**Table 1:** Characteristics of participants in the MSOC qualitative study.

**Characteristic**	**Intervention course (*n* = 8)**	**Standard-care course (*n* = 6)**	***P*-value**
Age (years) mean, (SD; range)	49 (11.1; 32–63)	51 (12.2; 35–67)	0.75
**Gender**, ***n*** **(%)**
Female	7 (87.5)	5 (83.3)	0.84
Male	1 (12.5)	1 (16.7)	
**Country**, ***n*** **(%)**
Australia	1 (12.5)	3 (50.0)	0.09
New Zealand	3 (37.5)	3 (50.0)	
USA	4 (50.0)	0 (0.0)	
**MS type**, ***n*** **(%)**
RRMS	6 (75.0)	4 (66.6)	0.51
SPMS	1 (12.5)	1 (16.7)	
PPMS	1 (12.5)	1 (16.7)	
TSD (years), mean (SD; range)	8.0 (11.0; 1–31)	11.0 (15.7; 1–37)	0.76
Missing, *n* (%)	1 (12.5)	0 (0.0)	

MS, multiple sclerosis; PPMS, primary progressive MS; RRMS, relapsing remitting MS; SD, standard deviation, SPMS, secondary progressive MS; TSD, time since diagnosis. Difference of variables between intervention and standard-care courses were assessed by T-test and Chi-squared test.

### Themes

**Figure 2:**
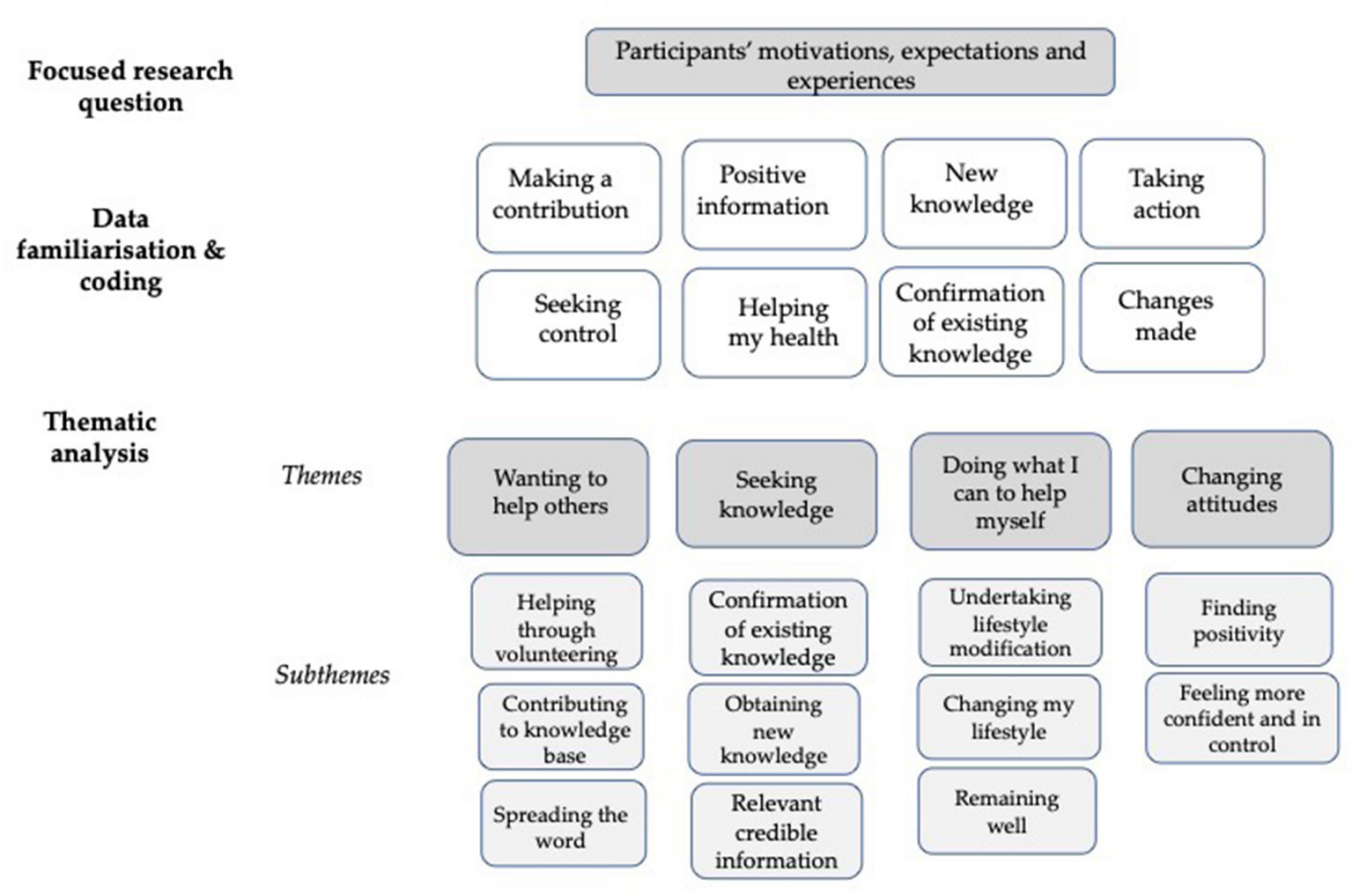
Diagrammatic representation of thematic analysis.

Four themes with subthemes were identified. A diagrammatic representation of the analysis, with a small representative sample of codes, is provided in Figure 2. Participants were identified as IC or SCC and an assigned research number.

#### Theme 1: Wanting to help others

The theme of wanting to help others identified some of the participants' motivations for being involved in the research project. Participants wanted to contribute to MS research and help others through any means they could. Subthemes included: helping through volunteering; contributing to knowledge base; and spreading the word.

##### Helping through volunteering

Many participants expressed the desire to contribute as a volunteer and wanted to take the opportunity to help others with MS and their families.

*First, I saw this as a volunteer opportunity. (IC4)*

Others specifically wanted to contribute by volunteering as a research participant. They did not have a firm idea of how they could help, but wanted to do whatever they could to benefit others.

*I guess because it was framed as a research project, and so I just assumed it was whatever experience I've had can effect and inform and help research then I'm happy to participate. But no, it wasn't actually for what I would personally get out of it. (SCC5)*

##### Contributing to knowledge base

Some participants wished to further MS knowledge and to make this information widely available through research to ensure others with MS benefited from having access to knowledge.

*I also feel like the more I can contribute to research and understanding of MS, the more MS patients are going to benefit. (IC4)*

##### Spreading the word

Some participants were motivated by wanting to learn more themselves so they could be a resource for others with MS, to be a support to peers and in that role, share personal experiences and insights as a way of helping others.

*If I can probably put across any sort of knowledge I have or any insight, any help I can give someone, I think that's, that's probably the best thing you can do, to be a resource for people. (SCC4)*

#### Theme 2: Seeking knowledge

This theme explored the many aspects of knowledge that were important to participants. The desire to obtain knowledge was a motivation to undertake the course. Participants had the hope and expectation that they would be provided with knowledge that would confirm, update and expand upon their existing knowledge. Participants also explored the experience of receiving credible, relevant and reliable knowledge and the impact that this knowledge had on them. Subthemes included: confirmation of existing knowledge; obtaining new knowledge; and relevant, credible information.

##### Confirmation of existing knowledge

Participants on the whole considered themselves relatively well-informed. For some, the information provided substantiation of knowledge they already possessed. They found confirmation of their existing knowledge comforting and reassuring.

*I think it being a confirmation of what I believe made me feel better. (IC8)*

Participants also wanted to have existing knowledge reinforced so they felt confident they were on the right path, doing as much as possible for themselves to manage their illness.

*I think I'm just permanently looking for people to reinforce that I'm doing the right things. (IC2)*

##### Obtaining new knowledge

Many were motivated by, and had the expectation of, receiving new knowledge to complement their existing knowledge. The MSOC provided a remedy for missed opportunities for learning that had occurred during the COVID-19 pandemic.

*I feel that I've missed out … I didn't get a lot of information because of COVID, like all the support groups were cancelled, all sorts of stuff. (IC7)*

However, some people in the standard care course felt the course didn't add much to their existing knowledge.

*I think it's about right. It may be a bit simplistic. (SCC2)*

##### Relevant, credible information

Participants had the expectation that they would receive relevant information. The introduction of modules by a person with MS, with whom participants could relate, helped to satisfy this expectation.

*It's nice to hear from someone who personally has experienced MS and then learn from their ways that they've dealt with the disease. So I thought that that was very effective. (SCC6)*

Participants expected and were impressed by the credibility of the sources of information, the presenters and the institution responsible for course development. For some the credibility assisted with their motivation to join the course.

*I felt I could access professional information, Melbourne Uni is something we hold in high regard. So I thought, well, I'll be on the cutting edge of what's going on with MS. (SCC1)*

Participants valued the science and evidence provided across both courses and the data provided helped make the information more credible and compelling.

*I liked the deeper look into the science behind it … I liked the statistics … I'm like a data person. So that's more compelling to me. (IC2)*

People valued the experiences of others that were presented within course videos. They found the sharing of outcomes people had achieved from undertaking lifestyle modifications inspiring and convincing.

*Seeing that she genuinely had had benefits from the exercise, I guess it was convincing sort of evidence. (SCC5)*

#### Theme 3: Doing what I can to help myself

This theme encompassed what participants hoped to understand regarding lifestyle modification, what they could implement so that they could help themselves, and some of the outcomes they experienced regarding making lifestyle changes following the course. Subthemes included: understanding lifestyle modification; changing my lifestyle; and remaining well and high-functioning.

##### Understanding lifestyle modification

Many participants were motivated to understand more about lifestyle modification. They wanted specific information to inform and validate the choices and to develop a greater understanding of how and why to make changes to help themselves.

*I want change and I want to understand and I certainly don't want to just be sitting at home feeling sorry for myself, and I want to know as much as I can about it to help myself. (IC2)*

The desire to undertake lifestyle modification was seen as an integral part of participants' taking responsibility for their own health. They wished to rely more on themselves and less on others.

*If I want to stay how I am now, wellness wise, I have to do something to keep it that way. I can't rely on somebody else to do it for me. I'm the only person who can make those changes. (SCC6)*

##### Changing my lifestyle

For some the knowledge they received translated into tangible behavior change. Some made changes that led to small but noticeable differences to their daily experiences.

*I'm making more of a conscious effort. If I don't need to use the processed meats, I won't. I won't eat cheese anyway. (SCC4)*

For others, especially those in the IC, the changes were more significant and immediate and there was an awareness of the impact of implementing changes.

*I made all the modifications to my diet. I took it really seriously and made the changes recommended … I will always stick to it now because of the impact that it's had. (IC6)*

*I did start following the diet. I was kind of like, following it, not following it. And then after I went to the doctor on Thursday, I said that's it. I really have to stop this because I have a five year old daughter and I don't want to be in a wheelchair when she goes to high school. So, overall, this was an amazing experience for me. (IC5)*

But for some, even in the IC, there was no real impact on the way they would manage their MS.

*Interviewer: Do you think that undertaking this course has had an impact? Participant: Probably not right at the moment. (IC3)*

##### Remaining well

Those who were well sought information to improve their functioning further. A participant in the SCC drew attention to the lack of information, that is publicly available and presented in the SCC, regarding the safety of hard physical exercise.

*It would have been nice at some point, if people had mentioned about high-functioning, like if you are high-functioning, you can continue to carry on being high-functioning. You're not going to do yourself any harm or damage by continuing to run or push yourself physically. (SCC6)*

#### Theme 4: Developing new attitudes

The theme of “developing new attitudes” explored some of the less well-defined hopes and expectations of participants and the more unexpected outcomes of undertaking the course. Subthemes included: finding positivity; and feeling more confident and in control.

##### Finding positivity

Participants described the wealth of negative messaging they commonly received, particularly online, about the future for people with MS. Some wanted to avoid being exposed to negative messages. They were actively seeking positive messages and hoped the course could provide these.

*I don't want to expose myself to that (negativity) because I want to continue to carry on with my life as is. So the online thing was a good way for me to filter… (SCC6)*

Some found that messages within the MSOC provided confirmation and reassurance which resulted in them feeling more positive, as a somewhat unexpected outcome.

*I think in a positive way, that some of the stuff I am doing, I know I'm doing it right, but it's always nice to hear other people say that's the way you should be doing it. (IC8)*

The information in the course provided a “wake up call” that resulted in a more positive attitude.

*[I feel] more positive because it was blatantly put to me, take care of yourself, stop. So, overall it was a positive experience. (IC5)*

##### Feeling more confident and in control

Some participants reflected on the loss of confidence and control they had initially experienced with their MS diagnosis. They wanted a greater sense of agency by having the tools to make changes to improve their health. Participants described how they were gradually able to restore their sense of confidence and control.

*I guess it does make me feel a little more capable and a little more responsible for the course of the disease. It makes you feel more confident in making those changes in your life. It was increasing my knowledge and making me more powerful in controlling my own life and my own disease. (IC4)*

There was a desire to increase control in self-managing MS, rather than to be a passive recipient of treatment.

*Wanting to learn as much as I can about MS and understand how I can help myself and be an agent for myself … as soon as I got a diagnosis, I felt like I lost control of my life. (SCC1)*

Taking medication and seeing healthcare professionals provided what participants viewed as intermittent help. They wanted to explore what they could do on a more continuous basis, every day, to gain control of their MS and maximize their outcomes.

*I am on a disease-modifying therapy that I take as an infusion once every six months…I wanted more information about actions I could take on a daily basis. (IC4)*

## Discussion

There is a paucity of studies examining perceptions of online learning in the context of MS. The aim of this qualitative analysis was to understand participants' motivations, expectations and experiences of participation in the MSOC feasibility study. The analysis identified four themes that provided insights into all three aspects of the research question, often simultaneously.

We identified multiple motivations for participation. Altruism, or “wanting to help others,” identified across both study arms, was a prime motivation. Participants described their desire to volunteer, help others, and be part of research to confer a wider public benefit. A systematic review of research participant motivation identified altruism as the second most common motivator, behind personal benefit (41) such as therapeutic interventions, not relevant in our participants. Other motivations included relevance of the illness and public benefit (42) and interest in the research topic (43), similar to themes expressed by our participants. In other observational research, altruistic motivation ranked highest among motivators, while intellectual, health-related, and financial motivations rated lower (44). Financial motivation was not relevant to our participants as no financial incentives were offered.

The theme of “seeking knowledge” provided insights into both motivations and expectations. Some were motivated by obtaining knowledge regarding lifestyle modification to improve their health, while others wanted knowledge to help maintain their current health and high levels of functioning. Several participants felt strongly that information for people who are well needed to be enhanced both in our course and on MS websites. People with MS identify that trustworthy information presented by people with MS is valued and prioritized (45) and seek information not driven by commercial interests, not provided by neurologists or medical websites, and related to practical and lifestyle-related information (46). Participants found the knowledge and course presenters in the MSOC credible, relatable and practical.

The theme of “doing what I can to help myself” is an uncommonly reported motivation to participate in research. More commonly, people wish to receive therapeutic benefits, closer monitoring, or access to new treatments (41), rather than learning about self-management. Our cohort did not explore direct personal benefit as a theme as the course did not provide therapeutic benefits or health monitoring. Subthemes explored some expectations of the course, such as hoping to be able to do something daily toward improving health.

Within the theme of “developing new attitudes,” several participants described the desire to develop positivity. To that end, they described a wariness and avoidance of negative messaging perceived as harmful to their wellbeing, and the importance of maintaining a positive, yet realistic perspective. Our participants found the MSOC to be positively presented and contributed to developing an attitude of positivity. Consistent with our findings, MS websites that greet users with negative descriptions of MS and present the worst health outcomes have been described by people with MS as having a negative impact on their emotional or psychological wellbeing (45). New attitudes also included a greater sense of control and agency, consistent with prior studies emphasizing the importance of self-efficacy in effective MS health promotion interventions (47, 48). An improved sense of control and confidence have also been identified in both people with MS (19), and their partners (49) who undertake a lifestyle modification workshop.

### Strengths and limitations

We acknowledge the potential for researcher unconscious bias in the development of the SSC and IC but the two arms appear to have been equally engaging as suggested by similar completion rates.

While the number (17/84, 20%) of people who participated in the MSOC feasibility RCT was relatively small, the proportion of course completers who participated in the qualitative interviews was high (14/17, 82%). As feasibility was achieved, non-completers of the course were not interviewed in this pilot trial. The views expressed are therefore those of those motivated to both undertake and complete the course.

Our sample was broadly representative of the MS community with a distinct female preponderance of 83% and 88% females in the SCC and IC study arms, respectively. The majority of participants across both study arms had RRMS with only one person in each study arm with PPMS and SPMS, respectively. Therefore, study findings largely represent people with RRMS. In addition, participants were recruited largely from research portals on MS websites meaning people highly motivated to contribute to research were likely to enroll, potentially influencing participants' motivations and expectations. Consequently, participants who expressly sought lifestyle modification information may have been positively disposed to the content of the course, influencing their perceptions and experiences.

However, we acknowledge that a major limitation of this study is the potential for selection bias as we only interviewed 17% (14/84) of people that expressed an interest in participating in the MSOC feasibility study, and 45% (14/31) of people who were randomized into the trial. While the proportion of people we interviewed is low, these figures are consistent with a prior qualitative study examining participants' experiences of a MS-related online course analyzing free-text responses from eligible participants, where 6.4% participation was observed (50). Further, while we were unable to correct for selection bias in our analyzes, qualitative research does not necessarily seek generalizability and this analysis provided rich data regarding these participants' experiences of the MSOC and is relevant given the overall paucity of qualitative studies examining participants' perceptions and impacts of online learning in the context of MS (51).

The MSOC and interviews were conducted during the COVID-19 pandemic and may have influenced both participant rates and the positivity with which the course was viewed due to limited alternatives.

## Conclusions

Semi-structured interviewing of a subset of people with MS participating in the feasibility study who completed the MSOC found participants' motivations to participate in the MSOC feasibility study included altruism, confirmation of existing knowledge, and the desire for new knowledge, including regarding lifestyle modification. Some participants reported making some modifications to lifestyle while others experienced improved confidence, positivity and sense of control. Findings may assist in targeting recruitment strategies and inform course redevelopment. Identification of participants' changing attitudes, which they considered a high priority in their lives, suggests outcomes should include measures of confidence and agency. Our findings also suggest that participants may implement the recommended healthy behaviors presented in the MSOC as has been evident in studies of participants in the residential workshop. An effectiveness study will lead to a further understanding as to whether the course may effect behavior change and improved health outcomes.

## Author's note

GJ is the author of Overcoming Multiple Sclerosis and co-author Recovering from Multiple Sclerosis, and the founder of the Overcoming MS Organization. GJ and SN were co-editors of Overcoming Multiple Sclerosis Handbook: Roadmap to Good Health and facilitators of OMS residential workshops for people with MS.

## Data availability statement

The University of Melbourne Human Research Ethics Committee requires that participants' anonymity is maintained. Participants were recruited on the basis that their data would not be shared other than via illustrative quotations.

## Ethics statement

This study was approved by the University of Melbourne Human Research Ethics Committee (ID: 1851781.2). The participants provided their written informed consent to participate in this study.

## Author contributions

SN, WB, JR, and PJ: conceptualization, methodology, and formal analysis. SN: resources, writing—original, supervision, and project administration. SN and WB: data curation. SN and GJ: funding acquisition. All authors: writing—revision and manuscript approval.
