# Supplementary material for: A multiple sclerosis lifestyle behavior online course: Qualitative analysis of participants' motivations, expectations and experiences
Source: Front Public Health. 2022 Dec 7;10:1022185. doi: 10.3389/fpubh.2022.1022185 (PMC9768550; doi:10.3389/fpubh.2022.1022185)
Supplement: Supplementary file 1 [file Data_Sheet_1.DOCX]

**Supplementary Figure 1. Semi-structured Interview Schedule**

1. What motivated you to undertake the course?
   1. Why did you want to take the course?
   2. Was there anything else you hoped to get from the course?
   3. Did the online nature of the course have any special attraction for you?
   4. Where did you hear about it?
2. What were your expectations of the course prior to starting?
   1. What were you hoping to get from the course?
   2. Was there something in particular you wanted to learn about?
   3. Was there something you wanted to understand better?
   4. Was there something you wanted to experience?
3. Tell me about your experience of the course?
   1. What parts worked well for you?
      1. Was there anything you wanted more of?
      2. What was the most important part of the course?
      3. What was the most important thing you learned?
   2. Did anything not work well, or was there anything you found difficult/would change?
      1. What do you think made it difficult?
      2. How could that have been different?
      3. Was there anything you wanted less of?
4. Do you think undertaking the course has had an impact on you and the way you see your future?
   1. What sort of impact?
   2. What things have changed?
   3. What about your motivation to change? Has that been influenced?
   4. How do you see your future? Has taking the course changed that at all?
5. We used to deliver this information in a face to face workshop. What are your thoughts about online delivery compared with face to face delivery?
   1. Would you have preferred to undertake it face to face?
   2. Did you use the forum? How did you find using the forum?
   3. Did you feel engaged with other participants?
6. What would you say to someone thinking about undertaking the course?
   1. How would you describe the course?
   2. Would you recommend the course?
7. Finally, is there anything else you want to say about your experience of the course?

*Prompts: can you say a bit more about that…give example?*
